# Equivalent Disease-Specific Survival Between Rural and Urban Osteosarcoma Patients: A Retrospective Analysis of the SEER Database

**DOI:** 10.3390/curroncol32040199

**Published:** 2025-03-28

**Authors:** Kate S. Woods, Mitchell A. Taylor, Peter T. Silberstein

**Affiliations:** 1 School of Medicine, Creighton University, Omaha, NE 68178, USA; mitchelltaylor1@creighton.edu; 2 Department of Internal Medicine, Division of Hematology and Oncology, Creighton University Medical Center, Omaha, NE 68124, USA

**Keywords:** osteosarcoma, cancer disparity, health equity, SEER

## Abstract

Osteosarcoma is the most common primary malignancy of bone. Previous studies have demonstrated rural-urban disparities in metastatic disease incidence and overall survival in high-grade osteosarcoma patients. However, there is a paucity of literature investigating disease-specific survival (DSS) disparities between rural and urban patients, which is explored herein using the SEER database. Patients with biopsy-proven cases of osteosarcoma were identified from 2000–2021. Statistical analysis was completed using SPSS version 29.0.2 and included chi-squared, Kaplan–Meier and log-rank, and stepwise Cox regressions. Statistical significance was considered at *p* < 0.05. Kaplan–Meier analysis revealed no significant differences in 5- and 10-year DSS between rural (55.0% and 47.0%) and urban patients (56.0% and 51.0%) (*p* = 0.107). Multivariable analysis further revealed no significant DSS difference between rural and urban patients (aHR: 1.03; 95% CI: 0.86–1.24; *p* = 0.757). This study expands upon prior research by investigating DSS between rural and urban osteosarcoma patients and finding no significant differences. While rural living is often associated with worse outcomes, important prognostic factors for osteosarcoma, including metastatic disease at presentation and tumor grade, were not significantly different between rural and urban patients in our study, possibly explaining our DSS-related findings. Factors other than geographical location likely impact outcomes, and future research should examine other ways that rural living may influence cancer care.

## 1. Introduction

Osteosarcoma, also known as osteogenic sarcoma, is the most prevalent type of bone cancer, accounting for approximately 20% of all bone sarcomas [1]. The majority of cases occur in children and adolescents, where nearly 75% of osteosarcoma cases are diagnosed in patients less than 25 years of age [2]. However, there is a bimodal age distribution in incidence, with a second peak occurring among the elderly aged greater than 65 [1]. Osteosarcoma tends to occur in the metaphysis of long bones, with the most frequent locations being the distal femur and proximal tibia [1]. Surgery was the initial primary treatment modality for osteosarcoma and was associated with poor survival rates of less than 20% [3]. It was not until the mid-1970s that outcomes began improving, when the utilization of chemotherapy caused a significant rise in prognosis [3]. Current neoadjuvant or adjuvant therapies have increased 5-year survival to approximately 70% in localized osteosarcoma [4]. High-dose methotrexate therapy (HDMTX)-based regimens are considered first-line chemotherapy agents to treat osteosarcoma and, while highly effective, are associated with serious side effects [5]. Thus, previous studies have investigated alternative methods, such as a cisplatin-ifosfamide-doxorubicin regimen, which has been shown to produce comparable outcomes to HDMTX-containing therapies with less adverse effects [5].

Despite advancements in treatment, osteosarcoma remains a challenging diagnosis and is often fatal given its aggressive behavior and potential for rapid metastasis [1]. Metastatic disease is the strongest predictor for osteosarcoma-related death [1,6], with metastasis to the lungs being the most common and highly predictive of survival outcomes [6,7]. Other factors, such as tumor grade and response to chemotherapy, are also important indicators of outcome in osteosarcoma [2,8]. In addition to disease-related factors, a number of demographic variables have been identified as prognostic factors in osteosarcoma, where patients of lower socioeconomic status groups have been shown to more frequently present with metastasis and larger tumors [6].

In recent years, there has been growing recognition of disparities in cancer care between rural and urban populations. Rural patients often face significant disparities in health outcomes due to factors such as limited access to primary care, specialty care, emergency services, and delayed seeking of care [9]. One study reported that among patients with high-grade osteosarcoma, rural patients were at a significantly increased risk for metastatic disease at presentation and reduced overall survival [10]. Previous literature has similarly highlighted rural disparities in disease-specific survival (DSS) for other malignancies, including the likes of melanoma [11], thymoma [12], and thyroid cancer [13]. However, there is a paucity of literature specifically examining DSS disparities between rural and urban patients diagnosed with osteosarcoma. Therefore, the aim of this study was to investigate whether rural-urban disparities exist in osteosarcoma-specific outcomes, using data from the Surveillance, Epidemiology, and End Results (SEER) database.

## 2. Materials and Methods

### 2.1. Data Resource

Patient clinicopathological and disease-specific survival data were collected using the SEER*Stat 8.4.4 software (National Cancer Institute/National Institute of Health, Bethesda, MD, USA) for the years 2000 to 2021. The Surveillance, Epidemiology, and End Results (SEER) database is a comprehensive population-based cancer registry that collects incidence and survival data from 17 cancer registries across the United States. These registries cover approximately 26.5% of the U.S. population and provide extensive demographic, clinical, and survival information on cancer patients. The SEER database is widely utilized for epidemiologic and clinical research due to its standardized data collection, broad geographic coverage, and long-term follow-up, making it a valuable resource for assessing cancer trends, treatment outcomes, and disparities in healthcare access. This study was deemed exempt from Institutional Review Board (IRB) approval, as SEER data is publicly available and de-identified, ensuring compliance with ethical and privacy regulations.

### 2.2. Population Selection

The SEER-17 database was queried to identify histologically confirmed cases of osteosarcoma diagnosed between 2000 and 2021. Cases were identified and selected using the International Classification of Disease for Oncology 3rd edition (ICD-O-3) histology codes 9180/3-9194/3, which represent various subtypes of osteosarcoma. Only tumors originating in primary bone sites were included, as defined by ICD-O-3 primary site codes C40.0-41.9. Patients with missing or unknown ICD-O-3 histology codes, primary tumor location codes, or survival data were excluded from analysis.

### 2.3. Variables and Outcomes

Epidemiologic factors including age at diagnosis, sex, race and ethnicity, annual income, and rural-urban living were collected. The classification of rural versus urban residence was based on the SEER Rural-Urban Continuum codes, which were originally developed by the Economics Research Service of the United States Department of Agriculture (USDA) [14]. Primary tumor location, tumor grade, subtype, and disease stage were recorded for disease characteristics. The SEER database utilizes a historical staging system that categorizes malignancies as localized, regional, or distant [15]. Localized stage is defined by SEER as a malignancy that has remained confined to its organ of origin without evidence of spread. The regional stage refers to a tumor that has extended beyond the boundaries of the primary organ or involves a regional lymph node. The distant stage is characterized by the metastasis of malignancy to remote areas of the body, either through direct extension, noncontiguous spread to distant organs or tissues, or dissemination via the lymphatic system to distant lymph nodes. This staging system applies to various types of malignancies and covers periods when formal staging systems may have differed. Patient vital status, disease-specific cause of death, and survival in months were also collected.

### 2.4. Statistical Analysis

Data were transcribed and analyzed using SPSS for Mac, version 29.0.2 (IBM Corp., Armonk, NY, USA). Frequency tables were generated to evaluate and describe baseline patient and disease characteristics. Associations between rural-urban living and categorical variables were assessed using chi-square and two-sided Fisher’s exact tests and Mann–Whitney U tests for continuous variables. Disease-specific survival (DSS) was analyzed using Kaplan–Meier survival curves to visualize survival over time for rural and urban groups. Differences in DSS between rural and urban patients were statistically assessed using the log-rank test. To further evaluate factors independently associated with DSS, a Cox proportional hazards model was constructed, adjusting for age at diagnosis, race and ethnicity, annual income, primary tumor location, disease stage, tumor grade, histologic subtype of osteosarcoma, and tumor size. Covariate selection for the multivariable model was performed using backward elimination to retain only significant predictors of disease-specific mortality. The proportional hazards assumption was validated by generating log-minus-log survival plots against the natural logarithm of survival time for each variable, confirming that no violations occurred and ensuring the validity of the model in estimating the independent effect of rural versus urban residency on disease-specific mortality. Statistical significance was considered at *p* < 0.05.

## 3. Results

A total of 5343 patients were identified from the SEER database (Table 1). The cohort consisted of 55.5% males and 44.5% females. The majority of patients (70.5%) were under the age of 40, with smaller proportions aged 40–49 (8.1%), 50–59 (7.0%), 60–69 (6.5%), 70–79 (4.8%), and 80 or older (3.1%). Regarding racial and ethnic distribution, 49.9% of patients were non-Hispanic (NH) White, 14.4% NH Black, 8.8% NH Asian or Pacific Islander (API), 0.6% NH American Indian or Alaska Native (AIAN), and 26.3% Hispanic of any race. Income distribution revealed that 47.1% of patients had an annual income below $75,000, while 52.9% had an annual income of $75,000 or greater. The vast majority (90.7%) of individuals resided within an urban setting, while only 9.3% were from rural communities. In terms of primary tumor location, 73.6% of cases originated in the limbs, 10.9% in the cranium, 9.8% in the pelvis, 3.0% in the thoracic region, and 2.7% in the spine. For tumor grade, 56.8% of tumors were undifferentiated (Grade IV), 30.1% were poorly differentiated (Grade III), 7.9% were moderately differentiated (Grade II), and 5.2% were well-differentiated (Grade I). Regarding disease stage, 37.1% of patients were diagnosed with localized disease, 39.0% of patients with regional disease, and 23.8% with distant-stage disease. The median tumor size of the entire cohort was 86.0 mm (interquartile range [IQR] 60.0–122.0). For treatment, the majority of patients received chemotherapy (78.0%), while 22.0% of individuals received no chemotherapy. Radiation therapy was less commonly utilized, with only 10.2% receiving treatment and 89.8% not undergoing radiation. Regarding surgery, 83.0% of patients underwent surgical intervention, while 17.0% did not receive surgical treatment.

Comparing rural and urban osteosarcoma patients, there was a significant association between rural-urban living and annual income (*p* < 0.001), where a greater percentage of urban patients had an annual income of $75,000+ (59.6%) compared to their rural counterparts (8.6%). Rural individuals were also more likely to be NH White (75.5% vs. 47.2%) and NH AIAN (1.8% vs. 0.5%), while a larger proportion of urban patients were NH Black (14.7% vs. 11.5%), NH API (9.5% vs. 2.2%), and Hispanic (28.1% vs. 9.1%) (*p* < 0.001). Urban patients were also more likely to be diagnosed at a younger age, with 71.0% of patients under the age of 40 compared to 65.3% of rural patients (*p* = 0.016), though there was no significant difference in sex distribution (*p* = 0.507). No significant differences were observed in primary tumor location (*p* = 0.094), disease stage at diagnosis (*p* = 0.694), histologic subtype of osteosarcoma (*p* = 0.645), tumor size (*p* = 0.692), or tumor grade (*p* = 0.601). However, differences in treatment were present, where rural patients were less likely to receive chemotherapy than urban patients (72.1% vs. 78.6%; *p* = 0.001), while rates of radiation therapy were similar (10.9% vs. 10.1%; *p* = 0.585).

**Figure 1:**
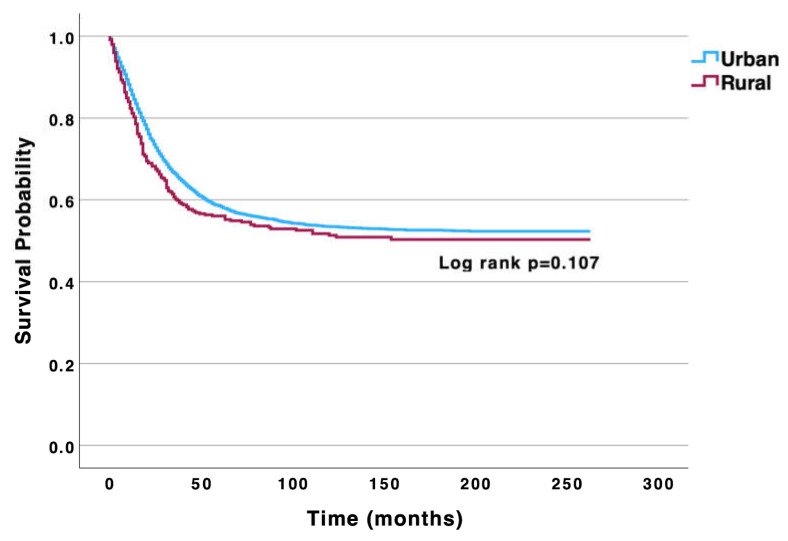
Univariable Kaplan–Meier analysis highlighting no significant difference in disease-specific survival between urban and rural osteosarcoma patients.

Kaplan–Meier DSS analysis of the entire osteosarcoma cohort revealed 5- and 10-year DSS rates of 56.0% and 51.0%, respectively. When comparing DSS rates between rural and urban patients, there were no significant differences in 5- and 10-year DSS rates between rural (55.0% and 47.0%, respectively) and urban patients (56.0% and 51.0%) (*p* = 0.107) (Figure 1). Furthermore, multivariable analysis adjusting for age, sex, race and ethnicity, annual income, primary tumor location, histologic subtype, disease stage at presentation, tumor grade, and tumor size further revealed no significant difference in disease-specific mortality between rural and urban patients (reference: urban; adjusted hazard ratio [aHR]: 1.03; 95% confidence interval [CI: 0.86–1.24; *p* = 0.757) (Table 2).

## 4. Discussion

Osteosarcoma is the most common type of primary bone malignancy, with metastatic disease, tumor grade, and tumor response to chemotherapy being important prognostic indicators of this disease [1,2,6,8]. Certain demographic factors, such as socioeconomic status and rural living, have also been shown to influence outcomes [6,10]. This study expands upon existing literature that has reported worse overall survival in patients with high-grade osteosarcoma residing in rural areas compared to those in urban areas [10]. In a retrospective analysis of the SEER database by Wendt et al., multivariable analysis revealed that patients residing within “very rural” locations independently experienced an increased overall mortality risk compared to “not very rural” patients after adjusting for metastases and tumor size (aHR = 1.58 [95% CI: 1.03–2.43], *p* = 0.037) [10]. According to their study, “very rural” patients consisted of individuals who resided in a rural county that was also not adjacent to an urban county. However, in the same study, the authors highlighted no increased overall mortality risk when comparing all rural patients to urban-dwelling patients (aHR = 1.34 [95% CI: 0.93–1.91], *p* = 0.112). Thus, our study adds additional insights by specifically investigating DSS between rural and urban osteosarcoma patients, reporting no significant differences in DSS. By focusing on DSS, our study allows for a more focused interpretation of treatment efficacy and how osteosarcoma patients are responding to current cancer care directed at their specific disease process.

It has been well established that rural patients often face worse outcomes and higher rates of comorbidities [16,17]. A report from the United States Department of Agriculture’s Economic Research Service revealed that age-adjusted natural-cause mortality rates were 6% higher in rural areas compared to urban areas in 1999, and this gap grew to 20% in 2019 [18]. Limited healthcare resources, physician shortages, delayed seeking of care, and a primarily older population were noted to be contributing factors to diminished survival in rural populations [9]. Another possible explanation for poor outcomes in rural patients is low socioeconomic status influencing access and quality of healthcare. Income is strongly tied to morbidity and mortality, and low-income Americans have higher rates of chronic conditions such as heart disease, stroke, and diabetes [19,20]. Low-income patients also are less likely to have health insurance, receive new medical treatments or technologies, and have easy access to primary or specialty care [20]. Our results demonstrate significant associations between rural living and an annual income of less than $75,000, yet rural status was not significantly associated with reduced DSS despite these socioeconomic disparities.

While multiple factors certainly contribute to overall health and outcomes in rural patients, our study suggests that rural living itself does not seem to play a significant role in disease-specific mortality in the case of osteosarcoma. These results remain true even when adjusting for potential confounding variables. Our analysis additionally revealed, however, that rural patients did not have significantly higher rates of metastatic disease at presentation, a factor known to be related to osteosarcoma-related mortality [1,6]. There was also no significant difference in tumor grade between rural patients and their urban counterparts, and low-grade osteosarcoma has been shown to have better outcomes than high-grade osteosarcoma [2]. Together, these findings help explain why our analysis showed similar DSS between rural and urban cohorts. Since key prognostic factors for osteosarcoma were not shown to significantly differ between rural and urban patients in our nationally representative cohort, it would be reasonable to expect that they share similar DSS outcomes.

An additional factor that has been shown to be predictive of prognosis in osteosarcoma is tumor response to chemotherapy [2,8]. The use of neoadjuvant or adjuvant chemotherapy with surgery for treatment of osteosarcoma has improved outcomes substantially by increasing 5-year survival rates, ameliorating symptoms and quality of life, and improving odds of full tumor resection [2,3,4]. Notably, our analysis revealed significantly lower rates of chemotherapy utilization in rural osteosarcoma patients compared to urban patients (72.1% vs. 78.6%, *p* = 0.001). Despite the recognized importance of chemotherapy in treating this malignancy, disease-specific mortality risk was not shown to be significantly different in rural individuals. As discussed previously, the value in measuring chemotherapy treatment as a prognostic factor lies in the response of the tumor to this treatment, not necessarily whether or not it was received [2,8]. Although the SEER database does not report any data regarding tumor response to chemotherapy, future studies investigating this could provide valuable insights into potential differences in chemotherapy response rates between rural and urban osteosarcoma patients.

Our findings are consistent with other studies demonstrating equivalent survival outcomes between rural and urban cancer patients. A multivariable analysis of the SEER database by Wang et al. investigating gastroesophageal neoplasms reported no significant differences in DSS between patients from large metropolitan areas compared to their rural counterparts for squamous cell carcinoma of the esophagus (aHR = 1.10 [95% CI: 0.91–1.33]), adenocarcinoma of the esophagus (aHR = 1.05 [95% CI: 0.93–1.20]), and adenocarcinoma of the gastric cardia (aHR = 1.07 [95% CI: 0.87–1.33]) [21]. Another study by Brungardt et al. demonstrated no significant difference in in-hospital mortality between rural and urban esophageal cancer patients undergoing esophagectomy (3.95% vs. 4.27%, *p* = 0.815; adjusted odds ratio = 1.64 [95% CI: 0.694–3.887], *p* = 0.300) [22]. These studies, in combination with findings from our study, collectively reinforce the notion that rural residency itself may not necessarily predict outcomes for all cancer types.

While our study suggests that rural residency alone may not independently contribute to DSS in osteosarcoma, it does not aim to discount the reality that rural living may impact other aspects of the disease course. Disparities between rural and urban communities are well documented across various aspects of cancer care, including quality of screening, treatment options, diagnostic and staging procedures, insurance coverage, health behaviors, and access to care [23]. Rural communities also face additional challenges due to limited public spending for social services that target social determinants of health, further leading to worse health outcomes [24]. Therefore, further research into the broader impact of rural living on cancer care is essential. These future studies could inform the development of strategies to overcome logistical barriers for rural patients, ultimately enhancing care for this underserved population. In the case of osteosarcoma, it would be prudent to address the disparities in chemotherapy receipt for rural patients, as demonstrated by our analysis. Thus, while our findings may not suggest that rural-urban differences are inconsequential, they highlight the need for a more comprehensive approach to understanding the multifaceted challenges rural patients experience when receiving cancer care.

Our study has notable limitations, including the retrospective nature of the SEER database, which limits causal inferences. Moreover, certain clinical and demographic variables are not represented within the SEER database, as it does not capture data on lifestyle factors, insurance status, or chemotherapy choices—elements that could potentially impact outcomes in osteosarcoma care. Further, the SEER database does not collect data on the type of treatment center where patients receive care, such as comprehensive cancer centers or academic facilities, nor data on distance to care centers, both of which may impact osteosarcoma care and outcomes. Nonetheless, these findings demonstrate comparable DSS rates between rural and urban osteosarcoma patients, suggesting that other factors may play a role in shaping outcomes. Future research should include longitudinal studies to assess long-term outcomes, such as disease recurrence and late-stage complications, in order to provide a more comprehensive understanding of disparities beyond survival outcomes.

## Figures and Tables

**Table 1:** Clinicopathological features by rural-urban living in an osteosarcoma cohort.

Total *n* = 5343	Urban (*n* = 4845)	Rural (*n* = 498)	*p*-Value
**Age at diagnosis (years)**			**0.016**
<40	3442 (71.0%)	325 (65.3%)	
40–49	398 (8.2%)	36 (7.2%)	
50–59	332 (6.9%)	41 (8.2%)	
60–69	304 (6.3%)	44 (8.8%)	
70–79	229 (4.7%)	29 (5.8%)	
80+	140 (2.9%)	23 (4.6%)	
**Sex**			0.507
Male	2684 (55.4%)	284 (57.0%)	
Female	2161 (44.6%)	214 (43.0%)	
**Race and ethnicity**			**<0.001**
NH White	2282 (47.2%)	375 (75.5%)	
NH Black	708 (14.7%)	57 (11.5%)	
NH API	460 (9.5%)	11 (2.2%)	
NH AIAN	25 (0.5%)	9 (1.8%)	
Hispanic (Any Race)	1355 (28.1%)	45 (9.1%)	
**Annual income ^∞^**			**<0.001**
<$74,999	1959 (40.4%)	455 (91.4%)	
$75,000+	2886 (59.6%)	43 (8.6%)	
**Primary tumor location**			0.094
Limbs	3534 (74.1%)	337 (69.2%)	
Cranial	516 (10.8%)	55 (11.3%)	
Spine	124 (2.6%)	19 (3.9%)	
Thoracic	141 (3.0%)	16 (3.3%)	
Pelvic	455 (9.5%)	60 (12.3%)	
**Disease stage**			0.694
Localized	1421 (37.0%)	148 (38.5%)	
Regional	1498 (39.0%)	151 (39.3%)	
Distant	921 (24.0%)	85 (22.1%)	
**Histologic subtype**			0.645
Fibroblastic	198 (4.1%)	20 (4.0%)	
Osteosarcoma, NOS	3279 (67.6%)	341 (68.5%)	
Chondroblastic	696 (14.4%)	64 (12.9%)	
Telangiectatic	147 (3.0%)	13 (2.6%)	
Osteosarcoma in Paget Disease of Bone	47 (1.0%)	2 (0.4%)	
Small Cell	40 (0.8%)	6 (1.2%)	
Central Osteosarcoma	137 (2.8%)	18 (3.6%)	
Intraosseous Well-Differentiated	14 (0.3%)	2 (0.4%)	
Parosteal	205 (4.2%)	22 (4.4%)	
Periosteal	53 (1.1%)	9 (1.8%)	
High-Grade Surface Osteosarcoma	29 (0.6%)	1 (0.2%)	
**Tumor size, mm (IQR)**	86.0 (60.0–122.0)	87.0 (60.0–121.5)	0.692
**Tumor Grade**			0.601
Well-Differentiated (I)	146 (5.1%)	19 (6.1%)	
Moderately-Differentiated (II)	224 (7.8%)	26 (8.4%)	
Poorly Differentiated (III)	858 (29.9%)	99 (32.0%)	
Undifferentiated (IV)	1641 (57.2%)	165 (53.4%)	
Chemotherapy *	3810 (78.6%)	359 (72.1%)	**0.001**
Radiation therapy *	486 (10.1%)	54 (10.9%)	0.585

Significant *p*-values (<0.05) are in bold. AIAN American Indian/Alaska Native; API Asian or Pacific Islander; IQR, Interquartile range; NH, non-Hispanic; NOS, not otherwise specified. SEER, Surveillance Epidemiology and End Results. * Compared to those who did not undergo this treatment. ∞ Annual income information was sourced from the United States Census Bureau’s 5-year estimates.

**Table 2:** Multivariable Cox regression identifying factors affecting disease-specific mortality in osteosarcoma patients.

Total *n* = 5343	aHR ‡	95% CI	*p*-Value
**Age at diagnosis (years)**			
<40	Reference		
40–49	1.63	1.34–1.98	**<0.001**
50–59	2.44	2.01–2.95	**<0.001**
60–69	2.93	2.41–3.54	**<0.001**
70–79	4.01	3.24–4.97	**<0.001**
80+	8.50	6.60–10.95	**<0.001**
**Sex**			
Male	Reference		
Female	0.86	0.77–0.95	**0.003**
**Race and ethnicity**			
NH White	Reference		
NH Black	1.17	1.00–1.36	**0.044**
NH API	1.01	0.83–1.23	0.920
NH AIAN	2.21	1.31–3.72	**0.003**
Hispanic (any race)	1.04	0.91–1.18	0.575
**Annual income**			
<$74,999	Reference		
$75,000+	0.84	0.75–0.94	**0.002**
**Rural-urban living**			
Urban	Reference		
Rural	1.03	0.86–1.24	0.757
**Primary tumor location**			
Limbs	Reference		
Cranial	1.06	0.88–1.27	0.545
Spine	1.79	1.36–2.36	**<0.001**
Thoracic	0.93	0.69–1.26	0.646
Pelvic	2.12	1.82–2.46	**<0.001**
**Histologic subtype**			
Fibroblastic	Reference		
Osteosarcoma, NOS	1.39	1.04–1.86	**0.028**
Chondroblastic	1.35	0.98–1.86	0.062
Telangiectatic	1.03	0.67–1.59	0.905
Osteosarcoma in Paget Disease of Bone	1.16	0.64–2.09	0.632
Small Cell	1.18	0.66–2.12	0.574
Central Osteosarcoma	0.91	0.59–1.42	0.691
Intraosseous Well-Differentiated	0.18	0.02–1.28	0.086
Parosteal	0.28	0.16–0.51	**<0.001**
Periosteal	0.65	0.32–1.32	0.233
High-Grade Surface Osteosarcoma	1.47	0.72–3.02	0.295
**Disease stage**			
Localized	Reference		
Regional	1.45	1.27–1.66	**<0.001**
Distant	4.67	4.07–5.36	**<0.001**
**Tumor grade**			
Well-Differentiated (I)	Reference		
Moderately-Differentiated (II)	1.06	0.57–1.99	0.851
Poorly Differentiated (III)	3.12	1.84–5.29	**<0.001**
Undifferentiated (IV)	2.99	1.77–5.03	**<0.001**
**Tumor size (cm)**	1.00	1.00–1.00	**<0.001**

AIAN, American Indian/Alaska Native; aHR, adjusted hazard ratio; API, Asian or Pacific Islander; CI, confidence interval. ‡ Adjusted hazard ratio refers to risk of disease-specific mortality after adjusting for important covariates. Inclusion of covariates into multivariable model was statistically determined using stepwise backward elimination. Significant *p*-values (<0.05) are in bold.

## Data Availability

The data utilized in this manuscript is available upon request from the corresponding author.

## Abbreviations

The following abbreviations are used in this manuscript:

DSS	Disease-specific survival
SEER	Surveillance, Epidemiology, and End Results
